# Prognostic value of the creatinine-albumin ratio in acute pancreatitis debridement

**DOI:** 10.1186/s12893-020-00991-6

**Published:** 2020-12-09

**Authors:** Zhifeng Zhao, Yeping Yu, Rongli Xie, Kaige Yang, Dan Xu, Li Li, Jiayun Lin, Lei Zheng, Chihao Zhang, Xin Xu, Ying Chen, Zhiwei Xu, Erchen Chen, Meng Luo, Jian Fei

**Affiliations:** 1 Department of General Surgery, Shanghai Ninth People’s Hospital Affiliated to Shanghai Jiao Tong University School of Medicine, No.639, Zhizaoju Road, Huangpu District, Shanghai, People’s Republic of China; 2 School of Clinical Medicine, Ruijin Hospital Affiliated to Shanghai Jiao Tong University School of Medicine, Shanghai, People’s Republic of China; 3 Luwan Branch, Ruijin Hospital Affiliated to Shanghai Jiao Tong University School of Medicine, Shanghai, People’s Republic of China; 4 Pancreatic Treatment Center, Ruijin Hospital Affiliated to Shanghai Jiao Tong University School of Medicine, No. 197, Ruijin No.2 Road, Huangpu District, Shanghai, People’s Republic of China; 5 Department of Emergency, Ruijin Hospital Affiliated to Shanghai Jiao Tong University School of Medicine, No. 197, Ruijin No.2 Road, Huangpu District, Shanghai, People’s Republic of China

**Keywords:** Acute pancreatitis, C-reactive protein, Creatinine, Albumin

## Abstract

**Background:**

Increases in the levels of serum C-reactive protein (CRP) and creatinine (Cr) and decreases in those of albumin (Alb) are commonly observed in acute pancreatitis (AP). We aimed to evaluate the efficacy of the Cr/Alb and CRP/Alb ratios in the prediction of surgical treatment effect in AP patients.

**Methods:**

This study retrospectively analyzed clinical data obtained from 140 AP patients who underwent debridement from January 2008 to November 2018 in Shanghai Ruijin Hospital. The Cr/Alb and CRP/Alb ratios at admission and before surgery were assessed in the analysis of clinical statistics, prediction of prognoses, and logistic regression analysis.

**Results:**

The admission Cr/Alb had the best predictive value of the four ratios. This value was significantly higher in patients with re-operation and those who died (*P* < 0.05) and was correlated with the Acute Physiology and Chronic Health Evaluation (APACHE II) score, admission CRP/Alb, preoperative Cr/Alb, and post-operative complications. The admission Cr/Alb could predict the risk of AP-related re-operation and mortality with sensitivities, specificities and areas under the curve of 86.3%, 61.7% and 0.824, and 73.4%, 81.3% and 0.794, respectively. At a cut-off value of 3.43, admission Cr/Alb values were indicative of a worse clinical state, including impaired laboratory test values, APACHE II scores, rates of post-operative complications and re-operation, and mortality (*P* < 0.05). In the logistic regression analysis, admission Cr/Alb values were independently related to the APACHE II score, post-operative renal failure, and mortality.

**Conclusion:**

Cr/Alb is a novel but promising, easy-to-measure, reproducible, non-invasive prognostic score for the prediction of the effect of debridement in AP patients.

## Background

Acute pancreatitis (AP) is a severe abdominal disorder characterized by sudden onset, rapid progression, and high mortality [1]. During AP treatment, debridement is often required among those with severe infection, peripancreatic necrosis, ineffective conservative treatment, and the use of minimally invasive approaches such as percutaneous catheter drainage [2]. However, due to the wide range of inflammation and surgical debridement and potential for severe infection, the surgical treatment of such patients is associated with a complication rate of 34–95%, re-operation rate of 60–70%, and perioperative mortality values of 11–39% [3, 4]. Considering the high risk of morbidity, mortality and poor prognoses during AP-related debridement, it is of great significance to evaluate and predict the risk of surgery before it is performed.

At present, several scores are used in the assessment of AP severity, such as Ranson’s Criteria and the Acute Physiology and Chronic Health Evaluation II Score (APACHE II). However, the calculation of those scores requires the use of numerous parameters and very complicated algorithms, due to which their use is restricted in clinical practice [5, 6]. Therefore, there is an urgent demand for a more simple, rapid and real-time tool for the prediction of disease severity and debridement efficiency.

Previous studies have shown that serum C-reactive protein (CRP), creatinine (Cr), and albumin (Alb) are related to AP severity and prognoses [7–12]. While their predictive value is unsatisfactory when used alone, their use, in combination, can effectively improve the sensitivity of prediction [13]. As the levels of CRP and Cr are often elevated, and those of Alb are often decreased in AP, a ratio including CRP or Cr and Alb could magnify the sensitivity of examination. CRP/Alb has previously been investigated in AP patients. At a cut-off value of 16.28, the mortality value in the higher CRP/Alb groups was 19.3 times that in the lower CRP/Alb group [14], suggesting its predictive value. Cr is also a valuable AP marker. However, no studies till date have investigated a ratio including Cr and Alb. Accordingly, this study aimed to explore the predictive value of Cr/Alb, and compare it with that of CRP/Alb in the assessment of surgical treatment in AP patients.

## Method

### Patients

A retrospective analysis was performed among AP patients who underwent surgery in Shanghai Ruijin Hospital from January 2008 to November 2019. Totally, 140 patients were enrolled in the study. All the procedures were implemented based on the principles of the Declaration of Helsinki, and the design of the work was reviewed and approved by the Ethics Committee of our hospital. As this is a retrospective research, the need for patient consent was waived by our institutional ethic committee.

The inclusion criteria were: (1) enrollment based on the Atlanta Classification criteria for AP that requires the presence of at least two of the following factors: (1) abdominal pain highly suggestive of AP, (2) elevations in the levels of serum amylase and/or lipase to more than three times the upper limit of normal, and (3) presence of radiological findings (ultrasonography or computerized tomography) characteristic of AP [15, 16]; (2) performance of at least one debridement procedure for AP at our hospital. The exclusion criteria were: (1) presence of benign and malignant pancreatic tumors; (2) incomplete medical records; (3) age < 18 years or pregnant; (4) combination with other digestive system diseases.

All AP patients were diagnosed based on the presence of at least two of the following three criteria: (i) abdominal pain suggestive of AP, (ii) elevation in serum amylase and/or lipase > 3 times the upper limit of normal, and (iii) computed tomography (CT) findings characteristic of AP. Presence of organ failure was assessed at admission and every 24 h during the hospitalization [15, 16]. After admission, the patients received standard treatment, including gastrointestinal decompression, anti-infection, pain control, inhibition of pancreatic secretion, and fluid resuscitation. Antibiotics were used empirically first, and then adjusted using the results of microbial body fluid cultivation. Surgical therapy followed the step-up principle, comprising anti-infection therapy, minimally invasive approaches, and laparotomy. All surgery-related decision-making was made through the discussion of a multiple disciplinary team comprising specialists from the Departments of General Surgery, Critical Care Medicine, Imaging, and Anesthesiology. All debridement procedures were performed by experienced surgeons from a specialized pancreatitis team.

### Data collection

Data were collected from the history system in our hospital. The laboratory examination values of CRP, Cr and Alb were collected separately at admission and within 24 h before the first debridement. Other variables included demographics, etiology, interventions, surgical complications, and outcomes.

### Definition

The presence of infected pancreatic necrosis was defined as purulent culture. Hemorrhage was diagnosed by a reduction > 2 g/dL and/or significant hemodynamic deterioration [17]. Shock was defined as a requirement for positive inotropic drug use or a systolic blood pressure < 90 mmHg. Respiratory failure was diagnosed as the ratio of arterial oxygen pressure and inspired oxygen concentration (PaO_2_/FiO_2_) ≤ 300 mmHg. Renal failure was defined as a serum Cr level > 176 μmol/L (2 mg/dL). Other complications were defined in accordance with the Atlanta Classification of AP [18].

### Statistical analysis

All statistical analyses were performed using SPSS 25.0. The Shapiro–Wilk test was used to evaluate the normal distribution of the data. Categorical variables were expressed as numbers and percentages, and examined by χ^2^ test and Pearson’s test. Numerical data were expressed as $\overline{{\text{x}}}$  ± s, and the presence of significant differences was examined by Student’s *t* test. Receiver operating characteristic (ROC) curves and areas under the curve (AUCs) were used to assess the predictive value for prognoses. Logistic regression was performed in the multivariate analysis for the evaluation of independent risk factors. In the statistical analysis, *P* < 0.05 was considered statistically significant.

## Results

### General clinical characteristics

**Fig. 1:**
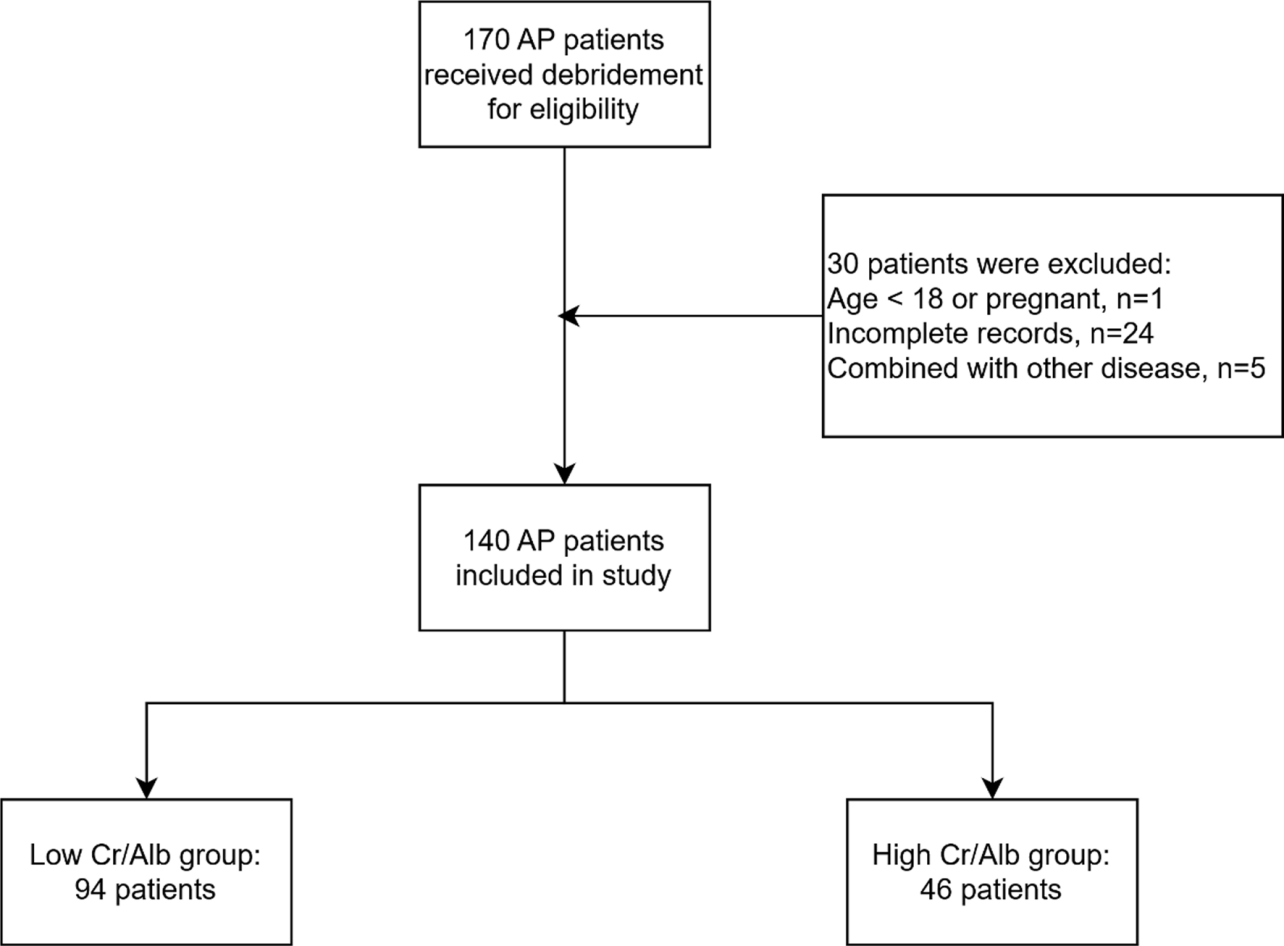
Flow chart of the research

After the exclusion of 31 patients, the study enrolled 140 patients (Fig. 1), including 98 men (70.00%) and 42 women (30.00%), with an average age of 49.88 ± 13.94 years. In terms of etiology, there were seven post-endoscopic retrograde cholangio-pancreatography cases, 66 gallstone cases, 28 hyperlipidemia cases, and 39 idiopathic AP cases. Of the 140 AP patients, 60 (42.86%) underwent re-operation, including 40 cases of debridement, 13 of hemostasis, and seven of debridement combined with hemostasis. Complications occurred after debridement in 90 patients (64.29%), including sepsis in 37 patients, electrolyte disorders in 31 patients, respiratory failure in 40 patients, hemorrhage in 34 patients, shock in 36 patients and renal failure in 34 patients. Finally, 16 patients died during the perioperative period (11.43%), as shown in Table 1.

**Table 1:** Demographic and clinical characteristics of AP patients

Variables	Patients (n = 140)
Age, years	49.88 ± 13.94
Male/female, no. (%)	98 (70.00)/42 (30.00)
Etiology, no. (%)	
Post-ERCP	7 (5.00)
Gallstone	66 (47.14)
Hypertriglyceridemia	28 (20.00)
Idiopathic	39 (27.86)
Re-operation, no. (%)	60 (42.86)
Debridement	40 (28.57)
Hemostasis	13 (9.29)
Debridement & hemostasis	7 (5.00)
Post-operative complications no. (%)	90 (64.29)
Sepsis	37 (26.43)
Electrolyte disorder	31 (22.14)
Raspatory failure	40 (28.57)
Hemorrhage	34 (24.29)
Shock	36 (25.71)
Renal failure	34 (24.29)
Outcomes, no. (%)	
Survival	124 (88.57)
Death	16 (11.43)

### Distribution of the Cr/Alb and CRP/Alb ratios in groups of re-operation and mortality

The admission CRP/Alb was only significantly higher in the re-operation group (*P* < 0.05). The distributions of the preoperative CRP/Alb values showed no significant differences between both subgroups (*P* > 0.05). The admission Cr/Alb was significantly higher in the re-operation group (*P* < 0.001) and mortality group (*P* = 0.011). The preoperative Cr/Alb was significantly higher only in the mortality group (*P* < 0.01), as shown in Table 2. The APACHE II score was significantly higher both in the re-operation and mortality groups, with *P* < 0.05, similar to the admission Cr/Alb. As a result, admission Cr/Alb and CRP/Alb and preoperative Cr/Alb were chosen for further analysis.

**Table 2:** Distribution of ratio variables in groups of re-operation and perioperative death

Variables	Re-operation group			Mortality group		
	Single-operation	Re-operation	*P* value	Survival	Death	*P* value
Admission CRP/Alb	2.90 ± 3.02	4.63 ± 2.80	0.005*	3.31 ± 2.88	4.98 ± 3.96	0.074
Admission Cr/Alb	3.07 ± 3.21	7.31 ± 7.57	< 0.001**	4.22 ± 5.21	10.11 ± 8.09	0.011*
Preoperative CRP/Alb	2.82 ± 2.92	2.50 ± 2.30	0.636	2.72 ± 2.86	2.53 ± 1.46	0.748
Preoperative Cr/Alb	3.83 ± 4.79	6.45 ± 8.27	0.147	3.59 ± 4.62	11.47 ± 10.17	< 0.01**
APACHE II	12.57 ± 6.55	18.80 ± 8.65	< 0.001**	14.89 ± 8.11	20.62 ± 7.24	0.016*

*Means *P* < 0.05, **means *P* < 0.01

### Correlation analysis between the ratios and general clinical statistics of the AP patients

Table 3 shows the correlation between the three ratios and the patients’ general clinical characteristics. The admission CRP/Alb was correlated with admission Cr (r = 0.243, *P* = 0.013), admission CRP (r = 0.959, *P* < 0.001), admission Alb (r = 0.284, *P* = 0.022), preoperative CRP (r = 0.295, *P* = 0.017), admission Cr/Alb (r = 0.241, *P* = 0.014), and intensive care unit (ICU) duration (r = 0.294, *P* = 0.003). The admission Cr/Alb was correlated with the APACHE II score (r = 0.495, *P* < 0.001), admission blood urea nitrogen (r = 0.815, *P* < 0.001), admission Alb (r = − 0.221, *P* = 0.009), admission Cr (r = 0.983, *P* < 0.001), preoperative Cr (r = 0.375, *P* = 0.002), admission CRP/Alb (r = 0.241, *P* = 0.014), preoperative Cr/Alb (r = 0.428, *P* < 0.001), and post-operative complications (r = 0.382, *P* = 0.002). The preoperative Cr/Alb was correlated with admission blood urea nitrogen (r = 0.326, *P* = 0.010), admission Cr (r = 0.412, *P* = 0.001), admission Cr/Alb (r = 0.428, *P* < 0.001), and ICU duration (r = − 0.254, *P* = 0.040). Of those ratios, admission Cr/Alb presented the greatest application value through its correlation with most of the eight variables, including the other two ratios, simultaneously.

**Table 3:** Correlation analysis between ratios and general clinical statistics of AP patients

Variables	Admission CRP/Alb		Admission Cr/Alb		Preoperative Cr/Alb	
	*r*	*P*	*r*	*P*	*r*	*P*
Age	− 0.143	0.149	− 0.107	0.209	0.137	0.276
Gender	− 0.044	0.729	0.068	0.590	− 0.009	0.943
BMI	0.230	0.051	0.073	0.495	− 0.067	0.645
APACHE II	0.176	0.093	0.495	0.000**	0.325	0.012
Admission WBC	0.142	0.171	0.174	0.051	− 0.061	0.648
Admission Hb	0.144	0.153	− 0.065	0.450	− 0.044	0.736
Admission TB	− 0.004	0.966	0.151	0.078	0.116	0.375
Admission BUN	0.087	0.391	0.815	0.000**	0.326	0.010*
Admission Alb	− 0.111	0.264	− 0.221	0.009**	− 0.186	0.140
Admission Cr	0.243	0.013*	0.983	0.000**	0.412	0.001**
Admission CRP	0.959	0.000**	0.182	0.083	− 0.026	0.845
Preoperative Alb	0.284	0.022*	− .046	0.716	− 0.121	0.337
Preoperative Cr	0.048	0.704	0.375	0.002**	0.984	0.000
Preoperative CRP	0.295	0.017*	− 0.123	0.330	− 0.126	0.318
Admission CRP/Alb	/	/	0.241	0.014*	0.005	0.971
Admission Cr/Alb	0.241	0.014*	/	/	0.428	0.000**
Preoperative Cr/Alb	0.005	0.971	0.428	0.000**	/	/
Post-operative complications	0.132	0.295	0.382	0.002**	0.230	0.065
ICU duration	0.294	0.003**	0.055	0.523	− 0.255	0.040*

*BMI* Body Mass Index, *APACHE II* Acute Physiology and Chronic Health Evaluation, *WBC* white blood cell, *Hb* hemoglobin, *TB* total bilirubin, *BUN* blood urea nitrogen, *Alb* Albumin, *Cr* creatinine, *CRP* C-reactive protein, *ICU* intensive care unit

*Means *P* < 0.05, **means *P* < 0.01

### Predictive value of Cr/Alb for re-operation and mortality

**Fig. 2:**
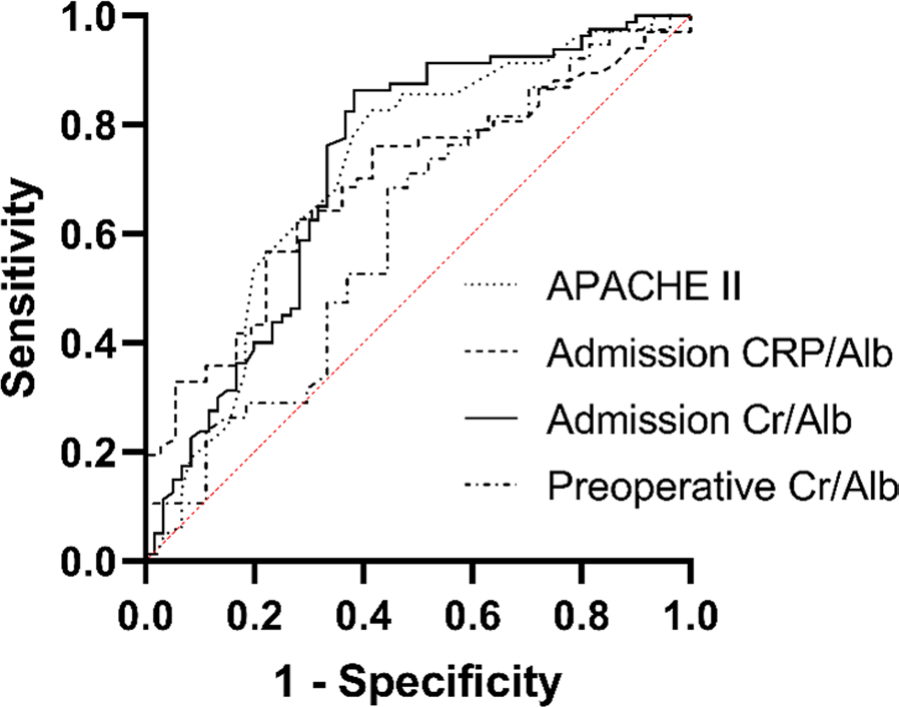
The ROC curve of multiple variables in assessing the re-operation of AP debridement which including APACHE II, admission CRP/Alb, Admission Cr/Alb and Preoperative Cr/Alb. Among them the Admission Cr/Alb had the highest AUC (0.724), APACHE II the second highest (0.716), Admission CRP/Alb the third highest (0.691) and Preoperative Cr/Alb the lowest (0.606)

**Fig. 3:**
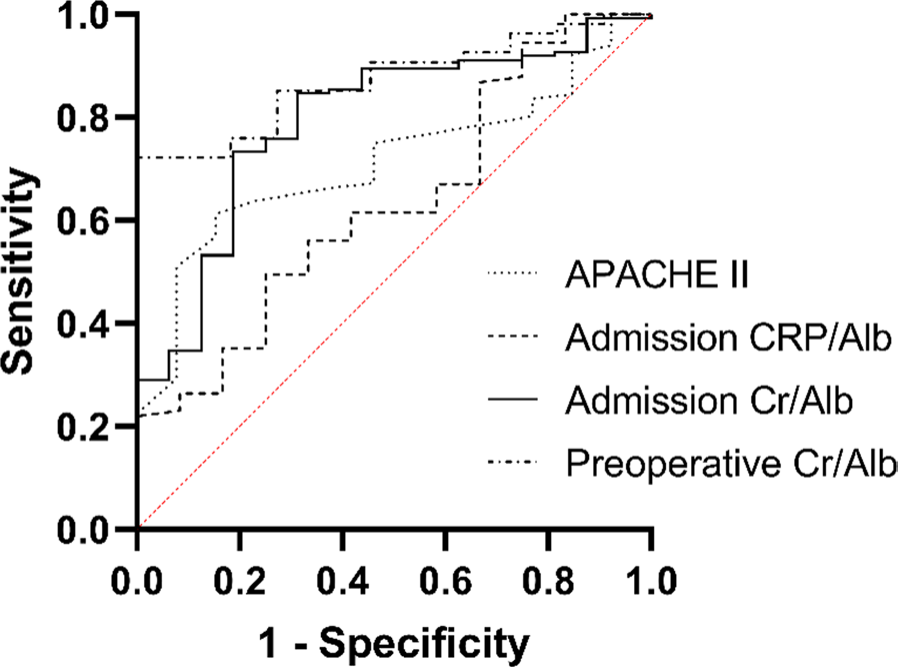
The ROC curve of multiple variables in assessing the mortality of AP patients which including APACHE II, admission CRP/Alb, Admission Cr/Alb and Preoperative Cr/Alb. Among them the Preoperative Cr/Alb had the highest AUC (0.872), Admission Cr/Alb the second highest (0.794), APACHE II the third highest (0.713) and Admission CRP/Alb the lowest (0.635)

Figures 2 and 3 show the ROC curve of the admission Cr/Alb in the prediction of re-operation and mortality. According to the ROC curve, we calculated the predictive values including cut-off values, sensitivities, specificities, and AUCs (Table 4).

**Table 4:** Predictive values of ratio valuables for re-operation and mortality in AP patients

Ratio	Re-operation				Mortality			
	AUC	Sensitivity (%)	Specificity (%)	Cut-off value	AUC	Sensitivity (%)	Specificity (%)	Cut-off value
Admission CRP/Alb	0.691	76.1	58.3	4.41	0.635	49.5	75.0	7.69
Admission Cr/Alb	0.724	86.3	61.7	3.29	0.794	73.4	81.3	3.43
Preoperative Cr/Alb	0.606	55.6	68.4	2.50	0.872	100.0	72.1	2.57
APACHE II	0.716	82.6	58.3	16.5	0.713	50.9	92.3	12.5

The admission CRP/Alb showed the lowest efficacy, with sensitivity, specificity and AUC values of 76.1%, 58.3% and 0.691 for re-operation, and 49.5%, 75.0% and 0.635 for mortality, respectively. The admission Cr/Alb had a better predictive value, with a sensitivity of 86.3%, specificity of 61.7%, and AUC of 0.724 for re-operation, and a sensitivity of 73.4%, specificity of 81.3% and AUC of 0.794 for mortality. The predictive value of the preoperative Cr/Alb for mortality was more meaningful, with a sensitivity of 100.0%, specificity of 72.1% and AUC of 0.872, with a cut-off of 2.57. Additionally, its predictive value for re-operation was also satisfactory, with sensitivity, specificity and AUC values of 55.6%, 68.4% and 0.606 respectively. In comparison, the sensitivity, specificity and AUC values of the APACHE II score were 82.6%, 58.3 and 0.716 for re-operation, and 50.9%, 92.3% and 0.713 for mortality, respectively. In general, the predictive values of the Cr/Alb ratio and APACHE II score were basically the same.

### Relationship between admission Cr/Alb and clinical characteristics

As suggested above, the cut-off admission Cr/Alb value was 3.29 for re-operation and 3.43 for mortality. The latter was chosen as the basis of grouping, as a ratio higher than 3.43 simultaneously satisfies two criteria. Of patients in the milder AP (n = 94) and severe AP (n = 46) group, those in the latter group with a Cr/Alb value ≥ 3.43 had a more intense heart rate and respiratory rate (*P* < 0.05), impaired laboratory data, including those pertaining to blood urea nitrogen, Cr, Alb, white blood cells, platelets and the APACHE II score (*P* < 0.05), higher morbidity values associated with post-operative complications, including electrolyte disorders, respiratory failure, hemorrhage, shock and renal failure (*P* < 0.05), and higher rates of re-operation and mortality (*P* < 0.001). Besides, the interval times between onset and debridement were shorter in the severe group but still exceeded 3–4 weeks, in keeping with the Atlanta Classification criteria for AP (Table 5). These results suggest that a cut-off admission Cr/Alb value of 3.43 has the potential to distinguish severe AP from milder AP.

**Table 5:** Baseline characteristics of AP patients sorted by ratio of admission Cr/Alb

Variables	Cr/Alb < 3.43 (n = 94)	Cr/Alb ≥ 3.43 (n = 46)	*P* value
Demographic characteristics			
Age, years	49.73 ± 13.00	50.17 ± 15.83	0.862
Male, no. (%)	62 (65.96)	36 (78.26)	0.136
BMI, kg/m^2^	23.92 ± 3.46	24.83 ± 3.95	0.271
Etiology, no. (%)			0.317
Post-ERCP	4 (4.26)	3 (6.52)	
Gallstone	47 (50.00)	19 (41.30)	
Hypertriglyceridemia	15 (15.96)	13 (28.26)	
Idiopathic	28 (29.79)	11 (23.91)	
History, no. (%)			
Hypertension	25 (26.60)	14 (30.43)	0.651
Alcohol	22 (23.40)	12 (26.09)	0.744
Smoking	21 (22.34)	11 (23.91)	0.770
Diabetes mellitus	8 (8.51)	8 (17.39)	0.242
Fatty liver	5 (5.32)	1 (2.17)	0.536
CAD	4 (4.26)	1 (2.17)	0.635
Clinical presentation			
Interval time^a^, days	56.23 ± 51.14	35.18 ± 37.13	0.016*
Heart rate, bpm	102.03 ± 20.38	116.45 ± 23.10	< 0.001**
Temperature, ℃	37.13 ± 0.74	37.27 ± 1.02	0.412
MAP, mmHg	61.18 ± 37.54	67.66 ± 34.13	0.319
Respiratory rate, bpm	22.98 ± 5.60	25.95 ± 7.55	0.023*
Laboratory data in admission			
Hemoglobin, g/L	107.06 ± 26.30	107.3 ± 32.06	0.964
BUN, mmol/L	5.64 ± 3.13	17.83 ± 9.76	< 0.001**
Creatinine, μmol/L	59.29 ± 21.11	277.50 ± 187.02	< 0.001**
CRP, mg/L	102.52 ± 73.29	121.57 ± 83.89	0.262
Albumin, g/L	29.00 ± 5.71	26.80 ± 5.30	0.030*
WBC, × 10^9^ cells/L	11.94 ± 5.39	14.33 ± 7.28	0.039*
Platelet, × 10^9^ cells/L	239.08 ± 114.78	155.30 ± 93.27	< 0.001**
TB, μmol/L	39.55 ± 58.64	51.12 ± 67.60	0.307
Score			
APACHE II	12.59 ± 6.76	21.21 ± 7.83	< 0.001**
Post-operative complications, no. (%)			
Sepsis	21 (22.34)	16 (34.78)	0.117
Electrolyte disorders	14 (14.89)	17 (36.96)	0.003**
Respiratory failure	13 (13.83)	27 (58.70)	< 0.001**
Hemorrhage	15 (15.96)	19 (41.30)	0.001**
Shock	12 (12.77)	24 (52.17)	< 0.001**
Renal failure	7 (7.45)	27 (58.70)	< 0.001**
Outcome, no. (%)			
ICU duration, days	58.75 ± 75.13	73.91 ± 64.24	0.251
Re-operation	25 (26.60)	35 (76.09)	< 0.001**
Mortality	3 (3.19)	13 (28.26)	< 0.001**

*CAD* coronary artery disease, *MAP* mean arterial pressure, *CRP* C-reactive protein, *TB* total bilirubin, *BUN* blood urea nitrogen, *APACHE II* Acute Physiology and Chronic Health Evaluation II

*Means *P* < 0.05, **means *P* < 0.001

^a^Interval time means the interval days between onset of AP and first debridement

### Logistic regression analysis of the admission Cr/Alb in AP patients

In the logistic regression analysis of the admission Cr/Alb in AP patients, the APACHE II score, sepsis, electrolyte disorder, respiratory failure, hemorrhage, shock, renal failure, re-operation and mortality were enrolled after the elimination of interference variables through colinear diagnosis. The results suggested that the APACHE II score, renal failure and re-operation were independently related to the admission Cr/Alb (*P* < 0.05), as shown in Table 6. This indicated that the admission Cr/Alb could predict the risk of re-operation and post-operative renal failure independently. AP patients with high admission Cr/Alb values had 4.331 times the chance of renal failure and 3.824 times the chance of re-operation. Besides, the admission Cr/Alb was correlated independently to the APACHE II score (*P* = 0.009), suggesting that the ratio could be a simpler substitute for the APACHE II score in clinical settings.

**Table 6:** Logistic regression analysis of admission Cr/Alb in AP patients

Variables	OR	95% CI	*P* value
APACHE II	0.911	0.851–0.977	0.009**
Sepsis	0.806	0.248–2.617	0.720
Electrolyte disorder	1.368	0.407–4.598	0.613
Respiratory failure	0.996	0.268–3.702	0.995
Hemorrhage	1.069	0.314–3.642	0.915
Shock	1.350	0.369–4.963	0.651
Renal failure	4.331	1.071–17.523	0.040*
Re-operation	3.824	1.071–13.655	0.039*
Mortality	2.548	0.381–17.021	0.334

*Means *P* < 0.05, **means *P* < 0.01

## Discussion

In this study, we found that the Cr/Alb ratio is a novel but promising, easy-to-measure, reproducible, non-invasive prognostic score that can be used for the prediction of the effect of debridement in AP patients.

AP is an emergency gastrointestinal condition, characterized by a rapid onset and poor prognoses, with mortality values of up to 40% [19]. As AP often causes severe systemic inflammation and necrosis in the abdomen, debridement plays an important role in its treatment. However, the effect of surgery is not satisfactory owing to the complexity of pancreatic or peri-pancreatic infections, multiple organ attack, and violent systemic inflammation, due to which a large proportion of patients experience numerous post-operative complications, re-operation or even death. Therefore, it is important to evaluate patients’ prognoses before surgery. Previous studies showed that CRP, Cr, and Alb had definite implications for systemic inflammatory response-related conditions such as AP [20, 21]; however, their predictive value, when used alone, is not satisfactory. Therefore, some studies combined those parameters for an enhanced prediction effect [14, 22].

In our study, of all the admission and pre-operation Cr/Alb and CRP/Alb values, the admission Cr/Alb showed the best performance in several analyses, including the distribution analysis and correlation analysis, and had the best predictive value. An admission Cr/Alb cut-off ≥ 3.43 could predict worse prognoses in AP patients, including a more severe heart rate and respiratory rate, impaired levels of blood urea nitrogen, Cr, Alb, white blood cells and platelets, APACHE II score, post-operative electrolyte disorders, respiratory failure, hemorrhage, shock, renal failure, re-operation rate, and mortality. In the logistic regression analysis, the admission Cr/Alb was independently correlated with the APACHE II score, renal failure and re-operation. About the diagnosis value of Cr/Alb, it is basically satisfying in predicting the outcomes of the AP debridement. In predicting the re-operation, the sensitivity, specificity, and AUC of the admission Cr/Alb reached 86.3%, 61.7%, and 0.724, respectively. And the variables of APACHE II were 82.6%, 58.3%, and 0.716, respectively. In the assessment of perioperative mortality, the sensitivity, specificity, and AUC of admission Cr/Alb were 73.4%, 81.3%, and 0.794, respectively. And those of APACHE II were 50.9%, 92.3% and 0.713, respectively. Above all, our research suggests that the admission Cr/Alb has the potential to predict AP-related prognoses as a simpler alternative to the APACHE II score.

The development of AP is often accompanied by systemic inflammation, sepsis, and multiple organ failure. Therefore, the prognoses of AP are closely related to the severity of inflammation, nutrition intake, negative nitrogen balance, and functional reserve [23]. Of the various tests, CRP, Cr and Alb are the most relevant examinations. CRP is synthesized by the liver and responds to inflammation within a few hours [24]. Owing to its short half-life and high sensitivity, it is often used for the detection and assessment of inflammation [7, 25, 26]. Alb is a negative acute phase reactant synthesized by the liver, and its expression decreases during inflammatory reactions. In previous studies, Alb was shown to be related negatively to the severity of inflammation, disease prognoses, and mortality in AP [20, 27]. Cr is an amino acid derivative produced by the metabolism of muscle tissues. It is filtered through the kidney and its levels are raised significantly in systemic inflammatory diseases [28]. An increase in the Cr level 48 h after admission is considered a marker of pancreatic necrosis, hypovolemia and renal insufficiency [11]. According to Wilkman et al. [29], increased Cr levels are independent risk factors for 90-day mortality in AP patients. Lipinski et al. [10] also reported that higher Cr values at admission and after 48 h were associated with a higher incidence of mortality.

In complicated disease like AP, a single biomarker usually could not provide accurate prognostic effect. As a result, more and more combinations of biomarkers were being applied recently. In AP, the most frequently used values were CRP/Alb, Neutrophil–lymphocyte ratio (NLR), Platelet-lymphocyte ratio (PLR), etc., which exceeded excellent diagnostic values in the assessment of AP. Suppiah et al. [30] discovered that the elevation of NLR at the first 48 h of admission was significantly associated with severe acute pancreatitis as a result of neutrophilia and lymphopenia during the systemic inflammatory response. The sensitivity and specificity of NLR to predict SAP achieved 63–90% and 50–57% respectively. PLR also showed good application value for mortality prediction with sensitivity of 73.3% and specificity of 99.22%. Furtherly, it was found that the combination of PLR and NLR had the highest AUC in evaluating the prognosis of AP, with similar predictive value among other scoring systems [31]. Besides, Kaplan et al. [14] discovered that the CRP/Alb ratio could predict the mortality of AP patients with sensitivity of 92.1% and specificity of 58.0%, while this ratio was also positively corelated with Ranson score, Atlanta classification etc. As mentioned above, those predictors reflected the severity of inflammatory status outstandingly. However, surgical intervention of AP means higher requirements for the surgical tolerance, higher risk of complications and mortality. Merely inflammatory factors might not be enough. Therefore, our study used Cr and Alb to focus mainly on the evaluation of surgical outcomes.

In our study, Cr/Alb showed better performance than CRP/Alb. CRP level fluctuations are more intensive in AP and may lead to measurement-related difficulties and inaccuracies in severity evaluation. In comparison, Cr level trends are more stable, lending them more credibility for use as predictors. Cr also has the ability to reflect the pancreatic necrosis, protein decomposition metabolism and renal function. In pancreatic necrosis, a large amount of toxic substances and inflammatory factors would be released and directly attacking the kidney, leading to aggravated renal injury [12]. And the process in turn weakens the renal clearance ability and resulting in decreased surgical tolerance. Besides, Alb reflects a body’s protein reserve more accurately. Several studies have given possible mechanistic explanations, (i) the inflammatory response produced by AP increases catabolism and tissue consumption; (ii) AP releases a large number of inflammatory factors such as interleukin-1, and interleukin-6 leads to a decrease in albumin biosynthesis in liver; (iii) during the stress response, vascular permeability increases and albumin penetration into the tissue space [20, 32]. In summary, Cr and Alb could assess the degree of inflammatory response, catabolism, and fundamental status. In AP patients with weaker status, serum albumin tends to decrease because of the impaired hepatic synthesis function, albumin exudation and increased consumption; and creatinine tends to increase because of the renal injury and pancreatic necrosis. The combination of the two markers could indicate the severity and prognosis of AP debridement more reliably and accurately.

This study is the first to compare admission and preoperative Cr/Alb and CRP/Alb values in the assessment of AP debridement. AP is a highly inflammatory and catabolic state that often leads to malnutrition. These features increase the risk of re-operation and mortality in debridement. Therefore, our study focused on surgical tolerance by examining the reserve capacity and nutritional status in contrast to previous studies that focused on severity of systemic inflammatory response. Patients with poor surgical tolerance usually face higher risk of re-operation and mortality, which requires more prudence in surgical decision-making. For surgeons, our study provides a simpler and more feasible tool for the evaluation of the basic state of AP patients before surgery-related decision-making. For ward managers, the Cr/Alb value could aid in the identification of high-risk patients in advance and remind medical staff to strengthen the degree of ward care targeted at those patients. As the Cr/Alb value could distinguish mild AP from its severe form, our results can be used by hospitals and health policymakers in the formulation of more efficient gradient treatment strategies for AP.

Our study has several limitations. First, its retrospective design may have led to a certain selection bias. Second, the study included only 140 cases enrolled from a single center, leading to insufficient reliability. Third, this study focused only on AP patients with debridement. In the future, we will include patients with non-surgical treatment for further investigation.

In conclusion, as a clinical scoring system with high accuracy and simplicity, the admission Cr/Alb value was superior in the assessment of the severity of AP with debridement. With the confirmation of our results in larger-scale investigations in the future, Cr/Alb has the potential to improve the quality of the AP risk scoring system and prognostic prediction.

## Conclusion

As a cheap, convenient and minimally invasive marker, Cr/Alb is an independent predictor of re-operation and mortality for AP debridement. The Cr/Alb ratio has good predictive potential for AP debridement patients and its prognostic value is comparable with other diagnostic scores.

## Data Availability

The datasets generated and analysed during the current study are not publicly available as the data are being used in next study, but are available from the corresponding author on reasonable request.

## Abbreviations

CRP: C-reactive protein
Cr: Creatinine
Alb: Albumin
AP: Acute pancreatitis
APACHE II: Acute Physiology and Chronic Health Evaluation
CT: Computed tomography
ROC: Receiver operating characteristic
AUC: Areas under curve
ICU: Intensive care unit
NLR: Neutrophil–lymphocyte ratio
PLR: Platelet-lymphocyte ratio
